# 1409. Understanding the Current Status and Barriers to Infection Prevention in Emergency Departments: A Survey of 71 Hospitals across the United States

**DOI:** 10.1093/ofid/ofad500.1246

**Published:** 2023-11-27

**Authors:** Laya Dasari, Julio Ma Shum, Eileen F Searle, Krislyn M Boggs, Olivia L Chen, Ashley Sullivan, Carlos A Camargo, Erica S Shenoy, Paul Biddinger

**Affiliations:** Massachusetts General Hospital, Cambridge, Massachusetts; Massachusetts General Hospital, Cambridge, Massachusetts; Massachusetts General Hospital, Cambridge, Massachusetts; Massachusetts General Hospital, Cambridge, Massachusetts; Massachusetts General Hospital, Cambridge, Massachusetts; Massachusetts General Hospital, Cambridge, Massachusetts; Massachusetts General Hospital, Cambridge, Massachusetts; Massachusetts General Hospital, Cambridge, Massachusetts; Massachusetts General Hospital, Cambridge, Massachusetts

## Abstract

**Background:**

Infection prevention and control (IPC) is especially challenging in the emergency care setting due to ever-increasing emergency department (ED) crowding, boarding of patients, increasing rates of staff turnover, and other factors.

**Methods:**

2022 IPC practices in the ED setting were queried between January and April 2023 using the National Emergency Department Inventories (NEDI)-USA survey, which is administered annually to all United States EDs. In a subset of EDs, the expanded survey assessed reported adherence to IPC policies, barriers to policy compliance, use of transmission-based precautions signage, disinfection of reusable medical equipment, use of personal protective equipment (PPE), hand hygiene (HH), staff education and training, and perceptions of safety concerning healthcare-associated infections (HAIs).

**Results:**

Of 289 facilities surveyed, 71 (25%) have responded as of April 17, 2023, with 83% (59/71) located in suburban/urban settings (Table 1). Although compliance with IPC practices was high with 73% (49/67) reporting > 80% compliance with HH (Table 2), notably, compliance with signage was identified as a significant gap, with 25% (18/71) of EDs reporting that they rarely or only sometimes post signs for patients who required them. This issue was more pronounced with patients placed in hallways or overflow treatment spaces, where, in applicable hospitals, 71% (25/35) of EDs never, rarely, or only sometimes post transmission-based precaution signs. Despite this, most ED leaders (67%, 47/70) felt neutral or did not perceive HAIs to pose a significant risk to patients relative to other patient safety issues in the ED (Table 3).

**Figure:**
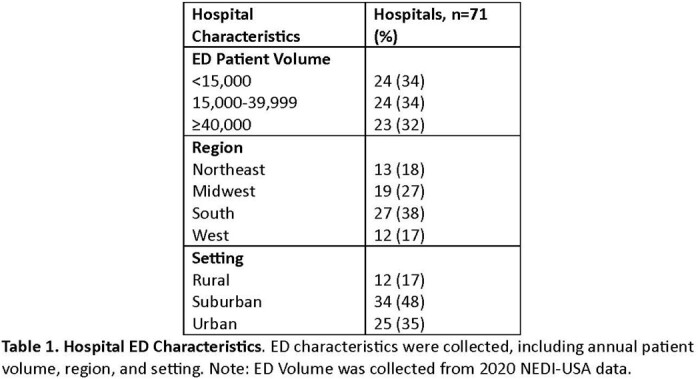

**Figure:**
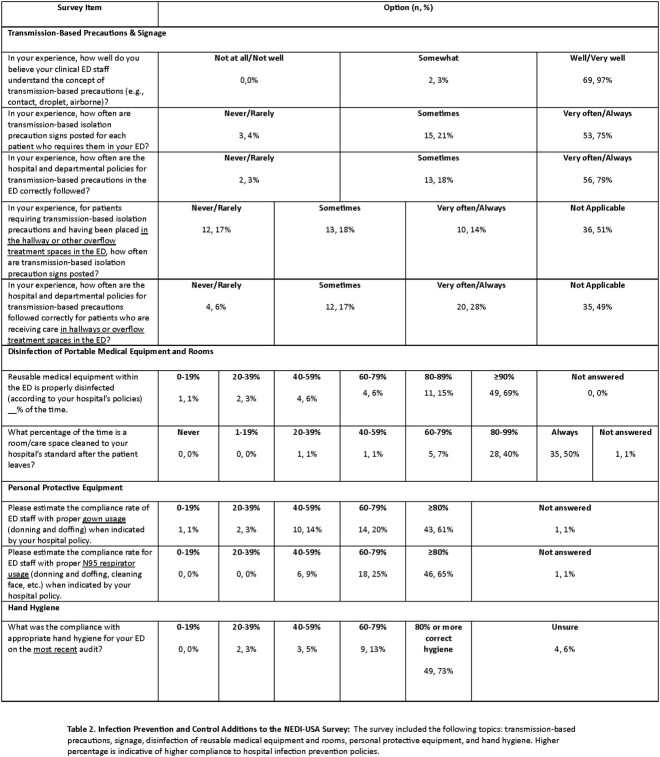

**Figure:**
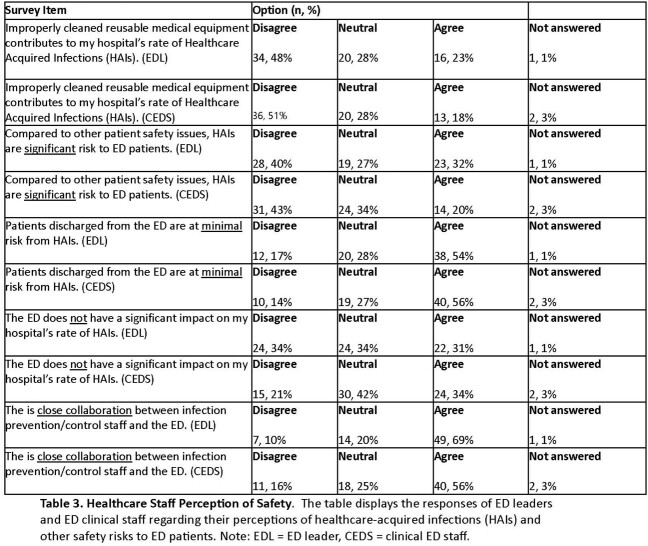

**Conclusion:**

This ongoing survey suggests high perceived compliance with IPC practices in the ED, which is discordant with evidence of suboptimal adherence to basic IPC practices. The findings suggest that targeted interventions and educational efforts are necessary to address the gaps in knowledge and understanding of IPC failures and risk of HAIs. The findings illustrate the importance of improving IPC practices in the ED to enhance patient safety; further research is needed to investigate the apparent discrepancy between perceived and actual practices.

**Disclosures:**

**All Authors**: No reported disclosures

